# Evaluation of the electronic Early Warning and Response Network (EWARN) system in Somalia, 2017–2020

**DOI:** 10.1186/s13031-022-00450-4

**Published:** 2022-04-16

**Authors:** Mutaawe Lubogo, Mary Joan Karanja, Rennatus Mdodo, Sherein Elnossery, Ali Abdirahman Osman, Abdulkadir Abdi, Evans Buliva, Muhammad Tayyab, Omar Abdulle Omar, Mirza Mashrur Ahmed, Solomon Chane Abera, Abdinasir Abubakar, Sk Md Mamunur Rahman Malik

**Affiliations:** 1 World Health Organization, Country Office, Mogadishu, Somalia; 2 World Health Organization, East Mediterranean Regional Office, Cairo, Egypt; 3 Federal Ministry of Health and Human Services, Mogadishu, Somalia

**Keywords:** Early Warning and Response Network, Integrated disease surveillance and response, Surveillance systems attributes, Somalia

## Abstract

**Background:**

In 2008, Somalia introduced an electronic based Early Warning Alert and Response Network (EWARN) for real time detection and response to alerts of epidemic prone diseases in a country experiencing a complex humanitarian situation. EWARN was deactivated between 2008 to 2016 due to civil conflict and reactivated in 2017 during severe drought during a cholera outbreak. We present an assessment of the performance of the EWARN in Somalia from January 2017 to December 2020, reflections on the successes and failures, and provide future perspectives for enhancement of the EWARN to effectively support an Integrated Disease Surveillance and Response strategy.

**Methods:**

We described geographical coverage of the EWARN, system attributes, which included; sensitivity, flexibility, timeliness, data quality (measured by completeness), and positive predictive value (PPV). We tested for trends of timeliness of submission of epidemiological reports across the years using the Cochran-Mantel–Haenszel stratified test of association.

**Results:**

By December 2020, all 6 states and the Banadir Administrative Region were implementing EWARN. In 2017, only 24.6% of the records were submitted on time, but by 2020, 96.8% of the reports were timely (p < 0.001). Completeness averaged < 60% in all the 4 years, with the worst-performing year being 2017. Overall, PPV was 14.1%. Over time, PPV improved from 7.1% in 2017 to 15.4% in 2019 but declined to 9.7% in 2020. Alert verification improved from 2.0% in 2017 to 52.6% by 2020, (p < 0.001). In 2020, EWARN was enhanced to facilitate COVID-19 reporting demonstrating its flexibility to accommodate the integration of reportable diseases.

**Conclusions:**

During the past 4 years of implementing EWARN in Somalia, the system has improved significantly in timeliness, disease alerts verification, and flexibility in responding to emerging disease outbreaks, and enhanced coverage. However, the system is not yet optimal due to incompleteness and lack of integration with other systems suggesting the need to build additional capacity for improved disease surveillance coverage, buttressed by system improvements to enhance data quality and integration.

## Background

Somalia, a country in the World Health Organization (WHO) Eastern Mediterranean Region (EMRO), has experienced a complex and prolonged emergency on an unprecedented scale. It is, therefore, graded by WHO as a protracted level 3 emergency country [1]. Three decades of civil unrest, conflict, natural disasters, including seasonal floods, droughts, and locust invasion, have contributed to the displacement of 2.6 million people, exposing them to recurrent disease outbreaks and health risks. A combination of multiple hazards, such as disease outbreaks, civil unrest, drought, cyclones and floods, has weakened Somalia’s health system, thus contributing to the poor health indicators, limited access to primary health care services, high levels of child and infant mortality compared to other countries in the region.

During emergencies, infectious disease epidemics are common. For example, based on data from multiple countries spanning a decade (1995–2004), during 63% of the 30 largest complex emergencies, infectious disease epidemics have been shown to have occurred compared to 23% of the 30 largest natural disasters [2]. Similarly, for Somalia, outbreaks such as cholera in 2017 undermined efforts to eradicate vaccine-preventable diseases such as polio and measles [3] by taking up the available healthcare workers who would otherwise have been engaged in healthcare, including vaccination. Additionally, outbreaks prevent health-seeking.Thus, not surprisingly, Somalia’s maternal and child mortality rates are among the world’s highest [4]. Simultaneously, lack of access to clean water in many areas has heightened the risk of outbreaks of water-borne diseases, augmenting existing vulnerabilities [5]. Basic healthcare infrastructure is fragile, and access to essential public health interventions is low.

In a fragile health situation like Somalia, timely detection of disease outbreaks is essential for appropriate public health response. WHO, health partners, and Ministries of Health across EMRO countries implement the Early Warning Alert and Response Network (EWARN) to detect and respond to alerts of selected epidemic-prone diseases [6]. Somalia is one of the countries under the Regional Office for East Mediterranean that implements the EWARN system for timely detection of diseases and public health response. The system is a network of health partners whose responsibility includes collecting and reporting surveillance data on priority epidemic-prone diseases, for early warning of disease outbreaks in humanitarian situations in emergencies where more comprehensive surveillance systems are not in place or are not functioning [7]. It has two main components: immediate reporting for alerts to signal cases of syndromes matching epidemic-prone diseases for further investigation and weekly reporting. The weekly reporting allows for reporting of data aggregated by health facilities. These complementary components ensure timely detection and verification of outbreaks and effective monitoring of morbidity patterns [8].

Since the establishment of EWARN in 2008, early warning surveillance in Somalia has faced technical and operational challenges that have hindered its functionality [9]. Following the collapse of EWARN system as a result of war, numerous vertical surveillance systems that included the polio surveillance system and the communicable disease surveillance and response system were established to fill the gap [10]. The CSR included five epidemic prone diseases for immediate reporting while nine disease events were for weekly reporting from a few sentinel sites that were accessible. In 2012, the list of reportable diseases was revised to include additional health events based on country needs and aligning the list of diseases to the 2005 International Health Regulation requirements (IHR-2005) [11].

EWARN collapsed between 2014 and 2015 due to civil conflict that led to the destruction of basic infrastructure and necessitated transferring the server from Mogadishu to Nairobi. In 2016/17, Somalia experienced a severe drought that led to drying up of water sources and limited availability of food which eventually led to displacement of people to camps searching for safe water and food. Limited access to safe water and proper sanitation among displaced populations led to cholera outbreaks in all districts. In the absence of a reliable surveillance system to support timely collection and response to cholera alerts, EWARN was reactivated by the Federal Ministry of Health (FMOH) with support from WHO in 2017 as a web-based electronic system [5]. EWARN is currently implemented in 689 sentinel health facilities in 118 districts of Somalia and uses a mobile application to report health alerts that are validated by surveillance officers at state level and data automatically analysed and displayed on an electronic dashboard. The goal is to have all health facilities in Somalia included in EWARN. Currently, majority are public health facilities, but there are also Non Govermental Organizations and major private health facilities reporting to EWARN. The health workers are trained and the facility indicates commitment to report regularly before a facility is added onto EWARN (URL:https://ewarn.emro.who.int/SOM/index.php/login.html). Standard guidelines and written standard procedures are available to guide EWARN performance [12]. EWARN serves an estimated population of 9.7 million people living in 6 states and the Banadir region of Somalia, of which 2.6 million live in internally displaced persons (IDP) camps and an estimated 30,000 as refugees. These people live in 118 districts, of which 35 (30%) are not accessible to humanitarian assistance and have low coverage of essential primary health care services.

EWARN is an adjunct, not a national disease surveillance system substitute. Once the acute emergency phase is over, it should be re-integrated into the national surveillance system [11]. In the initial implementation stages of EWARN implementation, a strategy for transitioning from EWARN to routine surveillance should have been clearly outlined [13]. However, it is critical to evaluate the performance of the surveillance system before transition or integration. This paper describes an evaluation of the performance of EWARN as the only surveillance system for real-time epidemic detection in Somalia after its re-activation in 2017. The article also describes the successes and challenges of implementing timely disease detection and response in a complex emergency and provides lessons that can be used to improve EWARN to fit into a more robust Integrated Disease Surveillance and Response (IDSR) system.

## Methods

### The EWARN system

As of 31 December 2020, the eEWARN system captured 15 diseases and health conditions, namely, severe acute respiratory infection (SARI), influenza-like illnesses (ILI), acute watery diarrhea/suspected cholera, bloody diarrhea/suspected shigellosis, other acute diarrhea, suspected diphtheria, suspected whooping cough, suspected measles, neonatal tetanus, acute flaccid paralysis, suspected acute jaundice syndrome, suspected viral hemorrhagic fever/Ebola, confirmed malaria, suspected meningitis, and COVID-19. With the SARS-CoV-2 pandemic in 2020, COVID-19 was added to bring the system’s number of diseases currently captured to 15. EWARN has 2 components, immediate and weekly reporting. Health facilities’ weekly data are aggregated and sent to the regional level by noon on Monday for each region’s surveillance officers’ approval. The verified data are then sent from the regions to the Ministry of Health (MOH) surveillance team by noon every Tuesday for final approval. MOH publishes the epidemiological bulletin after analyzing the submitted data every week on Thursday. The Emergency Coordinator generates EWARN reports at the FMOH office.

### Alert notification and weekly reporting

**Fig. 1:**
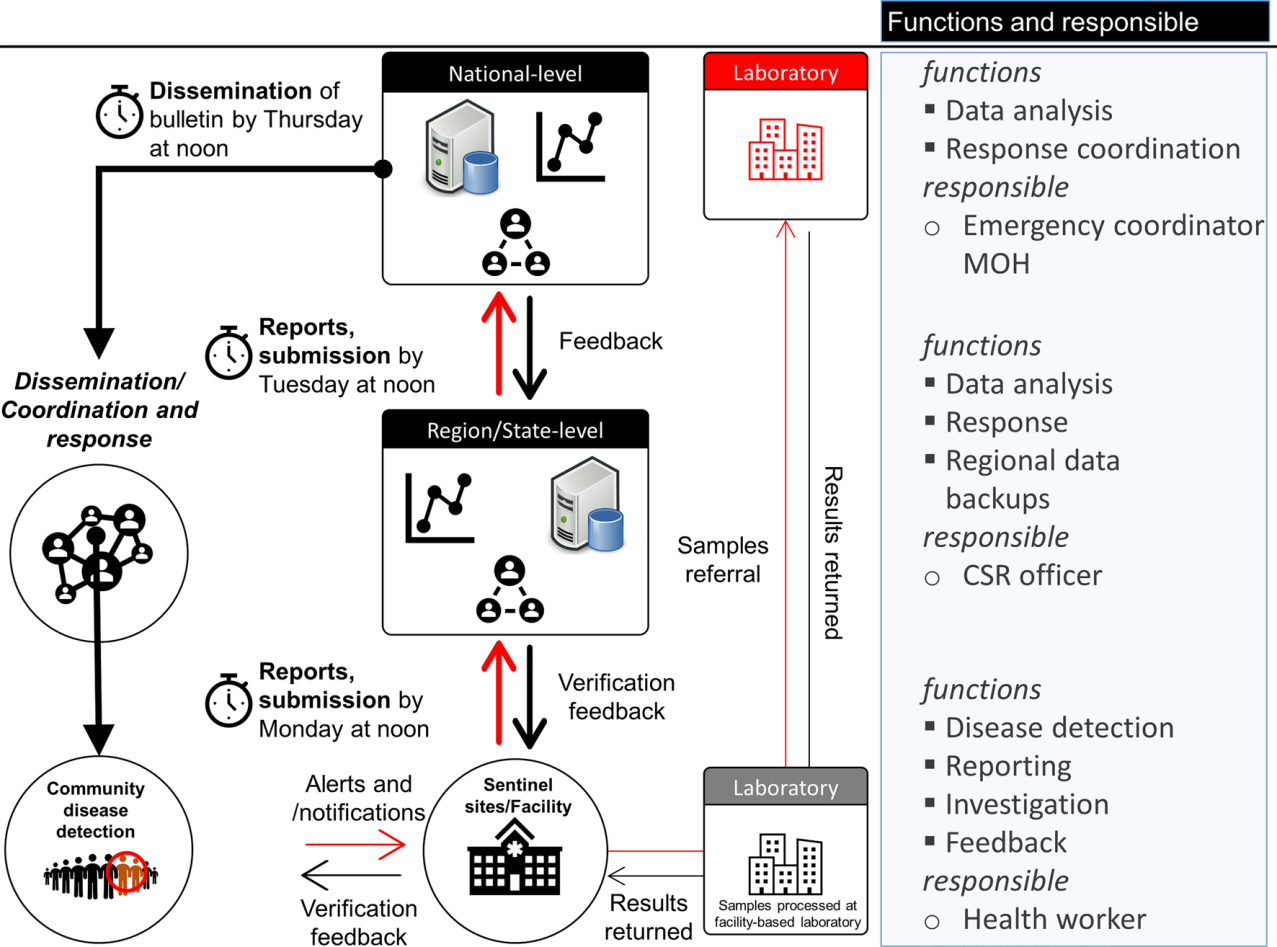
Data sources, flow, timelines, and responsibilities

Alerts of epidemic-prone diseases are reported from sentinel sites in different districts using a mobile application. Data from sentinel sites are immediately submitted to the regionally based surveillance officer for validation and instantly initiate alert investigation and verification with support from health partners (Fig. 1).

We used these expectations to determine timeliness, as described in the evaluation approach.

### Evaluation approach

Data for the period starting from week 1/2017 through to week 52/2020 were extracted from the eEWARN system using a spreadsheet and analyzed for quality attributes. We selected the review period due to data completeness. We used the standard attributes of a surveillance system and guidelines for evaluating a surveillance system, including a system such as EWARN [14]. For this evaluation, we focused on the following elements: a) *Sensitivity*, is the system’s overall ability to detect outbreaks, and monitoring trends of cases in a population over time. For this evaluation, we focused on the ability of the EWARN system to detect outbreaks; b) *Flexibility*, the ease with which a surveillance system can be adjusted to accommodate new diseases or new case definitions. For this attribute, we qualitatively evaluated the inclusion of new diseases to the system; c) *Timeliness*, the speed in weeks between two reporting steps in the eEWARN. Since the eEWARN reports are simultaneously submitted to the region and MOH levels, we calculated the timeliness of reports as the difference between the week when the reports were submitted and the week in which the diseases were detected. If the reports were submitted in the same epidemiologic week, we categorized reports submitted within the same week or just one week past the deadline as timely. In cases where the reporting was incorrectly documented this was categorized as discrepant. This attribute was assessed against a standard of at least 80%, which is the cut off for timeliness and completeness, of records submitted in a timely manner; d) *Data quality (measured by completeness)*, for this attribute, we simply compared the number of facilities that were reporting versus the expected; and e) *Positive predictive value (PPV)*, measured as the proportion of confirmed cases out of total alerts reported in EWARN. This attribute was assessed against a standard of at least 80% of EWARN sentinel facilities reporting.

As part of coverage assessment, we reported standardized rates of reported diseases per 100,000 consultations. Records extracted from the system with incomplete documentation were not considered in the analysis. We included data from health facilities that were reported between January 2017 and December 2020 in our analysis, which is the period following re-activation of the EWARN and the period with complete data.

### Statistical analyses

We measured eEWARN coverage, described system attributes, including PPV using proportions, and assessed the timeliness of reports in days and weeks while describing the distribution using median and interquartile ranges. We tested for trends of timeliness rates across the years 2017–2020 using the Cochran-Mantel–Haenszel stratified test of association. We carried out the analysis using Stata version 14.2 (Stata Corporation, College Station, TX). For all statistical tests, we used a p-value of < 0.05 to determine significance.

### Ethical considerations

EWARN is a surveillance system that does not include personally-identifying information. However, consent was obtained from the FMOH. Data are reported in aggregate form to inform public health response.

## Results

### Coverage

#### Sentinel sites coverage by state and populations

**Fig. 2:**
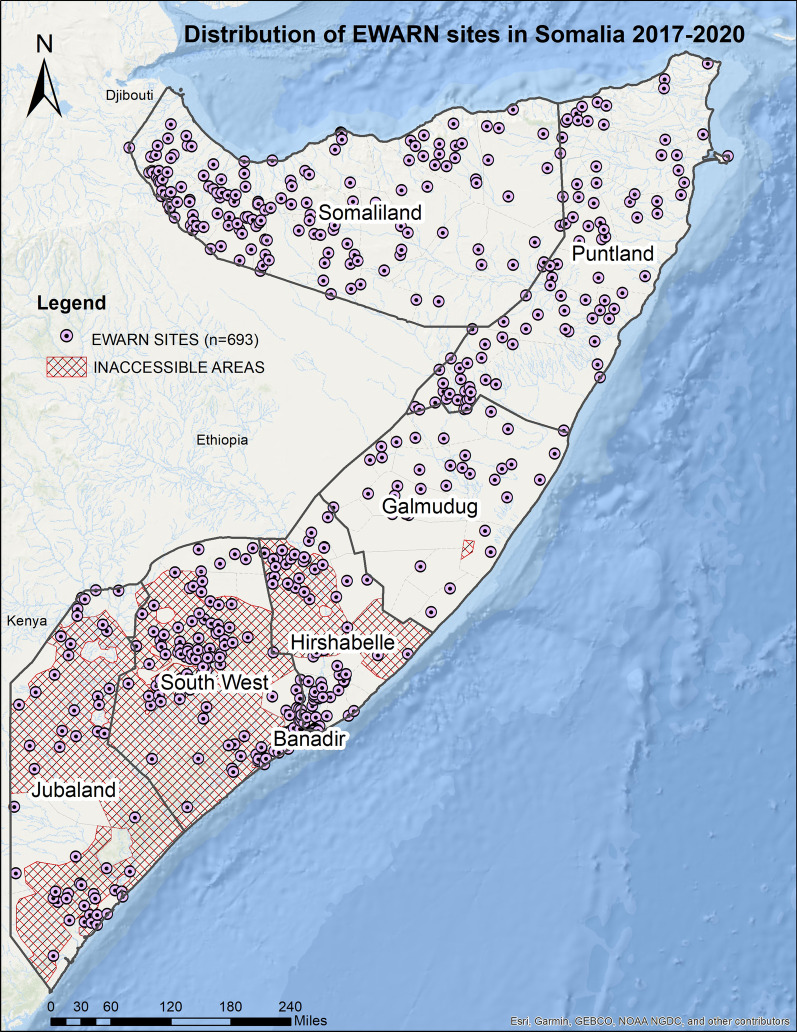
EWARN coverage Somalia 2020

As of December 2020, of the 1074 designated health facilities in Somalia, 689(64%) health facilities were actively reporting in EWARN. Most of the health facilities are located in Somaliland (28.0%), and the least are located in Galmudug (5.8%) (Table 1; Fig. 2). By 2020, an estimated 9.7 million people, including 1.1 million (9.0%) IDPs, were covered by the EWARN system. Most (24.0%) of the displaced populations are in Banadir, while the least (2.2%) are located in Somaliland (Table 1).

**Table 1:** Distribution of EWARN health facilities, population, and coverage by region, Somalia 2020

Region/state	Sentinel sites in 2020, n(%)^a^	Population	IDP (%)	Population coverage (n/N) (%)^†^
Banadir	87(12.6)	1,199,958	396,288 (24.0)	12.4
Galmudug	40(5.8)	655,791	119,768 (21.0)	6.8
Hirshabelle	66(9.6)	892,992	103,120 (9.9)	9.2
Jubaland	55(8.0)	767,415	134,328 (9.9)	7.9
Puntland	128(18.6)	1,785,984	129,387 (7.1)	18.4
Somaliland	193(28.0)	2,692,929	84,070 (2.2)	27.7
Southwest state	120(17.4)	1,716,219	166,790 (17.7)	17.7
Total	689	9,711,288	1,106,751 (9.0)	–

^a^These are sites reporting through the EWARN

^†^Coverage = the population/total population (excluding the IDPs)

#### Consultations per 100,000 population

The total number of consultations averaged 4 million annually for the period 2017–2020, with the highest number of total consultations of 4.7 million recorded in 2018 while the lowest number of total consultations recorded of 1.8 million people was reported in 2020. The most typical causes of morbidity reported in EWARN are ILIs (5121/100,000), acute diarrhea (5080/100,000), and severe acute respiratory illness (SARI) (18,690/100,000 population) (Table 2).

**Table 2:** Reported diseases in Somalia through eEWARN system, by rate per 100,000 population, 2017–2020

Reported diseases	2017	2018	2019	2020	All: 2017–2020
	n (rate^a^)	n (rate)	n (rate)	n (rate)	n (rate)
SARI	22,970 (627)	71,111 (1485)	94,978 (2499)	77,916 (4166)	266,975 (1890)
ILI	66,362 (1811)	146,400 (3056)	271,953 (7155)	238,710 (12,764)	723,425 (5121)
Cholera	12,568 (343)	6860 (143)	6709 (177)	9751 (521)	35,888 (254)
Shigellosis	2412 (66)	6642 (139)	8671 (228)	5456 (292)	23,181 (164)
Acute diarrhea	78,380 (2139)	168,872 (3525)	271,573 (7145)	198,844 (10,632)	717,669 (5081)
Diphtheria	299 (8)	364 (8)	391 (10)	502 (27)	1556 (11)
Pertussis	1044 (29)	3048 (64)	3253 (86)	2696 (144)	10,041 (71)
Measles	9337 (255)	9626 (201)	5764 (152)	4722 (253)	29,449 (208)
Neonatal tetanus	192 (5)	258 (5)	83 (2)	129 (7)	662 (5)
Acute flaccid paralysis	158 (4)	62 (1)	26 (1)	24 (1)	270 (2)
Jaundice	227 (6)	517 (11)	307 (8)	214 (11)	1265 (9)
Ebola	145 (4)	62 (1)	6 (0)	11 (1)	224 (2)
Malaria	14,188 (387)	20,160 (421)	29,717 (782)	17,978 (961)	82,043 (581)
Meningitis	249 (7)	1278 (27)	215 (6)	280 (15)	2022 (14)
COVID-19	0 (0)	0 (0)	(0)	1344 (72)	1344 (10)
Consultations	3,664,102	4,790,214	3,800,842	1,870,168	14,125,326

^a^Rate per 100,000 population in each year. Population data were from the Service Availability and Readiness Assessment (SARA), which included the catchment population for each facility[15]

### System attributes

#### Sensitivity

The EWARN system in Somalia detected occurrences of significant outbreaks, especially cholera and measles. Cholera and measles alerts were higher than other disease outbreaks in 2017. In 2017, there were two prominent spikes of reported cholera cases in May through June and again in September. For measles, the highest peaks were in November–December 2017 and February–March in 2018 (Fig. 2).

#### Flexibility

In response to emerging diseases and epidemics, the SARS-CoV-2 – Coronavirus disease (COVID-19) was included in the system. By the 52nd epidemiologic week of 2020, the eEWARN system had so far captured over 1300 cases (Table 2; Fig. 2).

#### Timeliness

The proportions of reports submitted on time from health facilities increased from 24.6% to the 96.8% in 2020 (p < 0.001).

#### Completeness

**Fig. 3:**
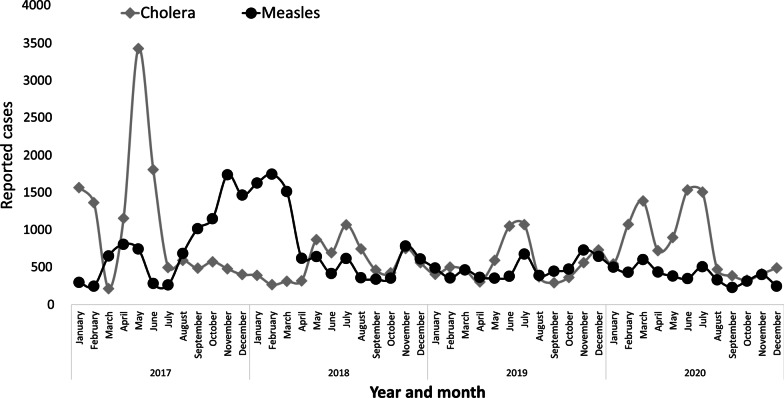
Trends of Cholera, and Measles reporting in Somalia in 2017–2020

Similarly, completeness (as an indicator of data quality) also improved from 31.5% in 2017 to the peak of 57.7% in 2019 before dropping to 55.7% in 2020. The number of health facilities reporting in EWARN also increased from 498 in 2017 to 689 in 2020. The year 2017 had the lowest overall report completeness with a median completeness of 26.3%, [interquartile range (IQR), (20.7, 49.0%)], completeness was best in 2019; median (58.5%, IQR 57.3, 60.0%) but deteriorated in 2020; median (35.6%, IQR 31.3, 35.5%) (Fig. 3).

#### PPV

The number of proportion of alerts verified increased from 2.0% in 2017 to 34.5% in 2020, p < 0.001 (Table 3). In the 4 years, the overall verification rate was 9.0%. The PPV increased from 7.1% in 2017 to a peak of 15.4% in 2019 and declined to 9.7% in 2020 (Table 3).

**Table 3:** Number of diseases detected, alerts, verifications, and positive predictive values in Somalia, 2017–2020

Year	Detected	Verified, n (%)	Alerts investigated	True alerts	PPV (%)
2017	10,617	211 (2)	42	3	7.1
2018	22,067	968 (4.4)	397	68	17.1
2019	22,068	968 (4.4)	117	18	15.4
2020	10,828	3731 (34.5)	236	23	9.7
Total	65,580	5878 (9.0)	792	112	14.1

## Discussion

### Coverage and expansion

**Fig. 4:**
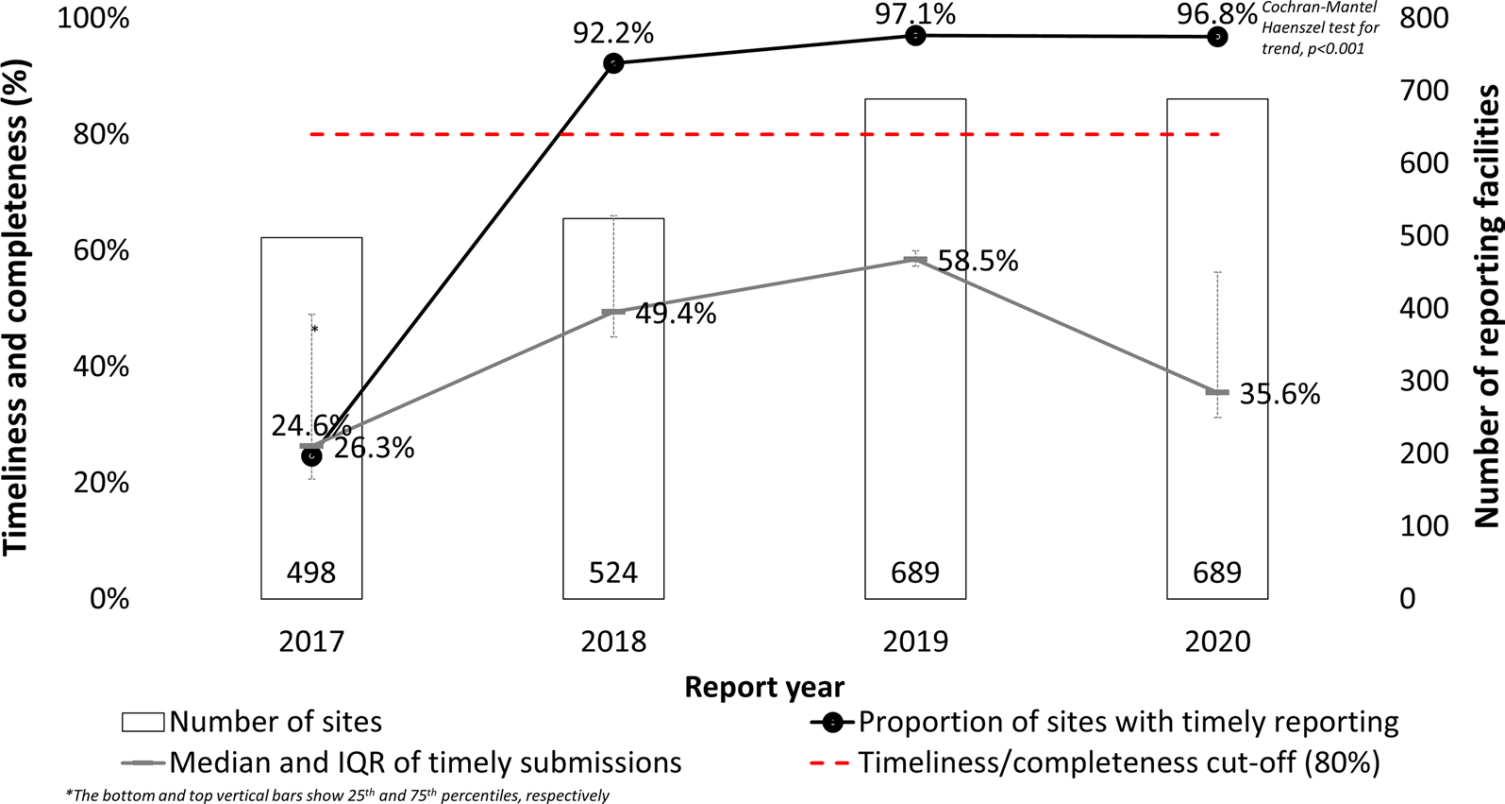
Timeliness and completeness of reports submitted through eEWARN by year, Somalia 2017–2020

Within the 4 years of implementation (2017–2020), coverage of the EWARN system in Somalia had covered 64% of all the health facilities despite a protracted conflict that has affected the country for the past 30 years.. In Somalia, as is the case in other countries, EWARN has been useful for the timely detection of alerts, monitoring disease trends and incidence for acute watery diarrhea/cholera, and 14 other health events, including COVID-19. In 2017, drought affected 118 districts of Somalia and led to severe water and food shortage forcing people to IDP camps. During this period, there was no reliable surveillance system in the country that would support the timely detection of alerts in Somalia’s remote districts. Despite the breakdown of telecommunication services and physical inaccessibility due to insecurity in different states of Somalia, timely submission of reports remained above average in all states. This success was achieved by providing mobile devices to health workers and airtime to support the submission of alerts of epidemic-prone diseases and enabled real-time submission of information according to set timelines in EWARN protocols. Thus, the digitalization of surveillance system processes through EWARN to replace the manual paper-based data collection that was time-consuming for health workers contributing to low reporting rates [16] proved to be useful as an expansion strategy. Somalia compares well with other countries in the East Mediterranean region with complex humanitarian situations since it is among the countries that achieved 64% EWARN coverage by 2019 [17]. However there is an observed uneven distribution of health facilities among regions with Galmudug, Hirshabelle and Jubbaland having the lowest coverage compared to other regions. Of the 118 districts of Somalia, 40 (33.9%) are inaccessible with all inaccessible districts being located in Jubaland, Galmudug and Hirshabelle regions (Fig. 4).

### Flexibility

The ability of a surveillance system to capture epidemics is critical. The system should detect changing prevalent disease patterns over time and detect and contain epidemics and public health threats of national and international importance [14]. Inclusion of new diseases and changes in case definition are critical elements for flexibility. In 2020, following the COVID-19 global pandemic outbreak, EWARN was expanded to capture and report cases of COVID-19. Flexibility is demonstrated when there is little or no additional cost in time, personnel, or funds involved [18]. The inclusion of COVID-19 as a reportable disease in Somalia utilized an already existing structure. Such rapid inclusion of COVID-19 in EWARN has also been reported in Syria [19].

### Sensitivity

Sensitivity is an equally important aspect of a surveillance system. The EWARN system was able to detect cholera spikes in 2017 and the overall changing patterns of diseases, including vaccine-preventable ones such as measles, tetanus, and acute flaccid paralysis (including polio). The cholera outbreaks in 2017 corresponded to the drought experienced in the same year. Although the EWARN system has demonstrated disease outbreak detection capability, such sensitivity ought to be interpreted alongside timeliness. For the EWARN system in Somalia, timeliness was at its lowest in 2017. Thus, the benefit of fast response may not have been achieved. Such situations where timeliness may undermine sensitivity are not uncommon, especially in times of war [20].

### Timeliness

The timeliness of reports is critical for public health response. To meet the timeliness threshold, in Somalia, reports need to be submitted to the national level by Monday of the week that follows the epidemiological week and by Tuesday of that same week to the Ministry of Health. The findings show that timely reporting increased by nearly two-fold from 2017 to 2019, and though it dropped slightly in 2020, timeliness was still at well over the 80% expected threshold. Improvement in timeliness may reflect the training of RRTs. Despite these improvements, there were some discrepancies between the reports’ entry dates and the epidemiological report week when diseases occurred. These discrepancies may indicate insufficient data entry checks or a lack of quality control measures within the eEWARN system. However, just like any other electronic surveillance system, the issues are not uncommon since such systems are characterized by inadequate data completeness, timeliness, quality, analysis and use, and lack of data integration from sources other than health care facilities [21]. In another publication, Somalia is indicated to have had a timeliness rate of 60% and thus is not listed among the 5 countries within EMRO that had achieved ≥ 80% reporting timeliness [17], possibly due to different methods used to analyze this indicator.

### Completeness

The EWARN system has had a steady improvement in the completeness from the lowest rates in 2017 when the EWARN system was getting established. However, completeness rates doubled by 2019 but declined sharply in 2020. This decline in 2020 could have been due to the COVID-19 pandemic. Completeness is an aspect that may be hard to measure due to various interpretations, such as those that emanate from disparities between individual-level and aggregate data [22]. Besides, the completeness of an integrated system such as EWARN needs to be compared across other disease-specific systems. However, our findings for 2019 show a completeness rate of 68% and are comparable with those of Mala et al. which rated Somalia to have a completeness rate of 70% [17].

### PPV

The study showed that since 2017, more than 1.8 million health alerts have been detected by the EWARN system, even though only 14% of alerts reported and investigated by WHO-supported surveillance officers and rapid response teams (RRTs) were authentic. This low PPV could be attributed to the low sensitivity of case definitions for health alerts reported in EWARN. Additionally, high staff attrition of trained personnel, especially in remote areas, may have contributed to the low PPV. PPV is an essential attribute of a surveillance system that starts with increased disease verification, the ability to detect diseases correctly. The EWARN system has been on an upward trajectory in disease verification, the number of illnesses verified, and coverage across the regions. In Somalia, the reported PPV is comparable to PPV for specific reportable diseases such as measles, reported being sub-optimal in Uganda, averaging < 10% [23].

### Limitations

Interpretation of the findings presented in this manuscript ought to be done with an appreciation of the EWARN system’s weaknesses in Somalia. Despite its success, the EWARN system in Somalia has faced many challenges that have negatively affected disease surveillance and response implementation. The protracted insecurity led to the obliteration of communication infrastructures, such as the system server’s destruction, affecting data completeness for previous years. Our analyses have therefore been limited to the period with complete data spanning 4 years. The telecommunication infrastructure, including internet connectivity services in Somalia, is limited to major towns making it challenging to collect timely information in remote health facilities. Hence, completeness may be problematic.

The high turnover of health workers from sentinel sites negatively affects completeness and timeliness. Reporting rates are also affected by a decrease in the number of active facilities over time. For example, the number of health facilities in Galmudug decreased from 60 to 40 due to many health facilities’ closure following the drought/famine/cholera. As seen in other states, expansion was due to the addition of new health facilities. Also, because health services are concentrated in urban areas, there is lower coverage of EWARN in rural areas. Most of the sentinel facilities are owned by NGOs with limited interest in EWARN due to competing interests. In an aggregate data reporting system such as EWARN, it may not be possible to duplicate reported events. Thus, an assessment of completeness and precision would be useful. The degree to which the data are precise could not be determined in this study.

## Conclusions and recommendations

Implementation of EWARN in Somalia faced significant challenges which negatively affected timely detection and response to health alerts. Limited funding from government and donor agencies contributed to the slow expansion against the set plans and the low number of alerts being investigated and verified by district based rapid response teams. The high staff turnover, especially from remote districts, created gaps in health facilities leading to late or lack of immediate and weekly surveillance reports. Interruption of telecommunication services by insecurity significantly contributed to the late submission of alerts in the EWARN system.

In the absence of other surveillance systems in Somalia, EWARN has played a crucial role in disease detection, verification, triggering the investigation, and reporting of diseases of public health importance in the country in real-time. The sharing of relevant health information with health partners and stakeholders to guide response activities and monitor these diseases’ trends across the country is equally critical. For its potential to be appreciated, improvements to the system would be necessary. Firstly, appreciating the importance of timeliness, the system should be modified to capture timeliness by diseases and not only by an epidemiological week. Our findings support the notion that for countries experiencing complex humanitarian crises, implementation of EWARN can provide timely information for monitoring disease incidence, trends, and evaluation of public health programs [10]. For successful implementation, there is a need to standardize protocols and training materials to support the EWARN system remotely where access is limited for better quality data. For overall improved usability, enhancements to the online platform and streamlining functionality should be considered. Finally, to improve EWARN coverage, there is a need to scale-up implementation to all health facilities focusing on underserved rural communities. Besides, integration with a community-based disease or public health event reporting system would be useful.

Although the EWARN approach has shown its worth, there remains a need to improve case identification, recording, and reporting. In general, performance indicators should be reviewed and strengthened. EWARN was designed for crises; however, Somalia’s delicate situation may necessitate its prolonged existence and expansion due to its adaptability, coverage, and acceptance. However, Somalia needs to consider repurposing the human resources currently supporting EWARN as one of the strategies for transition and integration into IDSR.

## Data Availability

The datasets used and/or analyzed during the current study are available from the corresponding author on reasonable request.

## Appendix 1: List of priority diseases/conditions for weekly surveillance, case definitions

*Selection based on the following criteria: 1) *epidemic-potential disease,* 2) *associated with high mortality*, and 3*) diseases for which intervention e*xists in Somalia—vaccine, vector control, hygiene promotion, etc.† *Note:* neonatal tetanus only meets criteria 2 and 3, suspected hemorrhagic fever moved to immediate alerts.

**Table:**

Diseases-suspected and confirmed	Case definition
Suspected cholera^a^/Acute watery diarrhea	Person aged 5 years or more with severe dehydration OR death from 3 or more acute watery stool per day (24 h), with or without vomiting
	Child aged 2–4 years with severe dehydration OR death from acute watery diarrhea, with or without vomiting
Suspected shigellosis/bloody diarrhea	Person with 3 or more loose stools (diarrhea) per day (24 h) with visible blood OR any person in whom a clinician suspects shigellosis
Suspected measles	Person with fever AND generalized, spotty (maculopapular, non-vesicular) rash AND ONE of the following: cough, runny nose (coryza) or red eyes (conjunctivitis) OR any person in whom a clinician suspects measles
Acute flaccid paralysis	Child younger than 15 (14 or less) with acute sudden onset of weakness or inability to move an arm or leg (flaccid paralysis) OR any person in whom a clinician suspects polio
Suspected diphtheria	Person with hoarse or complete loss of voice (laryngitis) or sore throat (pharyngitis or tonsillitis) AND non-removable coating (adherent membrane) of back of throat (tonsils or pharynx), and/or nose
Suspected whooping cough (pertussis)	Person with cough lasting at least 2 weeks AND ONE of the following signs:
	Fits of coughing (paroxysms)
	Making whooping sound when breaking in (inspiratory whooping)
	Vomiting immediately after coughing without other cause (post-tussive vomiting)
Confirmed malaria	Person with fever or history of fever (> 38.0 °C) in last 48 h and/or other symptoms AND positive laboratory confirmation by microscopy or rapid diagnostic test
Neonatal tetanus	Neonate aged 3 to 28 days with normal sucking and crying for first 2 days of life AND now cannot suck normally OR becomes stiff with jerking of muscles
Influenza-like-illness (ILI)	An acute respiratory illness with onset during the last 7 days with:
	Measured temperature ≥ 38˚C, AND
	Cough and/or sore throat (in the absence of a known cause other than influenza)
Other acute diarrhea	Any person with acute diarrhoea (passage of 3 or more loose stools in the past 24 h) with or without dehydration, and which is not due to bloody diarrhea or suspected cholera with or without dehydration, and which is not due to bloody diarrhea or suspected cholera

^a^Case definition for cholera, available at: http://www.emro.who.int/sudan/pdf/Cholera_CaseDefinition.pdf

## Appendix 2: List of syndromes for alert notification, case definitions

Daily alerts—from any source: community informants or leaders, religious leaders, traditional healers, non-health NGOs, media rumours, the affected population, healthcare facilities of any types, etc.

**Table:**

Diseases/syndromes	Alert thresholds	Case definitions
Suspected cholera	1 case where not previously	Use case definitions for weekly surveillance
Suspected shigellosis	5 cases in same week in the same community	
Suspected measles	1 case	
Acute flaccid paralysis	1 case	
Suspected diphtheria	1 case	
Suspected whooping cough	5 cases in same week in the same community	
Confirmed malaria	To be determined by weekly trends	
Neonatal tetanus	1 case	
Suspected meningitis	2 cases in same week in the same community^a^	Person with sudden onset of fever (> 38.0 °C at armpit) AND ONE of the following signs:
		Stiff neck when trying to move it (neck stiffness)
		Confused or difficult to wake up (altered consciousness)
		Bruising (hemorrhagic or purpuric) rash
		Child less than 1 year, with fever AND bulging of soft spot in top of head (fontanel)
Suspected viral hemorrhagic fever	1 case	Person with severe illness without confirmed malaria (negative laboratory confirmation) AND acute onset of fever (> 40.0 °C) AND ONE of the following signs:
		Bloody or black tarry stools
		Vomiting blood
		Bruising (hemorrhagic or purpuric) rash
		Coughing up blood
		Bleeding from other sites (eyes, nose, gums, skin, vagina)
Acute jaundice syndrome syndrome	5 cases in same week in the same community or health facility	Person with acute onset of yellowing of whites of eyes, inside of mouth, or skin or dark urine (jaundice) with or without fever AND no other cause, like excessive alcohol
Severe acute respiratory infection (SARI)^b^	5 cases in same week in the same health facility or hospital, *if present*	Person with a severe acute illness onset within the last 7 days with
		History of fever or measured fever (> 38.0 °C)
		Cough, AND
		*Requires* hospitalization
		NOTE: *Requires* italicized because hospitals may not be present in Somalia
Rumour of unusual cluster of deaths	3–5 deaths in same week in the same community or health facility	Persons who die suddenly of unknown, non-injury cause with same signs or symptoms and do NOT have any of the diseases or syndromes listed above
Rumour of an unusual event	2 or more cases in same week in same community or health facility	Persons with signs or symptoms that do not have any of the other alert diseases or syndromes

^a^2013 Revision: Alert threshold was simplified for easier reporting (previous threshold: 2 cases in same week in the same community [if camp/community population > 30,000: 5 cases/100,000/week]).

^b^2013 Revision: Case definition for SARI updated as per World Health Organization guidelines: WHO Interim Global Epidemiologic Surveillance Standards for Influenza. July 2012.

## Abbreviations

CDC: US. Centers for Disease Control and Prevention
COVID-19: Corona virus disease 2019
CSR: Communicable diseases surveillance and response
eEWARN: Web-based electronic Early Warning Alert and Response Network system
EMRO: Eastern Mediterranean Regional Office
EWARN: Early Warning Alert and Response Network
FMOH: Federal Ministry of Health
IDP: Internally displaced persons
IDSR: Integrated Disease Surveillance and Response
MOH: Ministry of Health
NGOs: Non-governmental organizations
PPV: Positive predictive value
RRTs: Rapid response teams
WHO: World Health Organization
